# Disseminated intravascular coagulation is associated with poor prognosis in patients with COVID-19

**DOI:** 10.1038/s41598-024-63078-9

**Published:** 2024-05-30

**Authors:** Satoshi Gando, Takayuki Akiyama

**Affiliations:** 1 Department of Acute and Critical Care Medicine, Sapporo Higashi Tokushukai Hospital, N34, E14, Higashi-ku, Sapporo, 065-0033 Japan; 2 Division of Acute and Critical Care Medicine, Department of Anesthesiology and Critical Care Medicine, Faculty of Medicine, Hokkaido University, Sapporo, Japan; 3 Large-Scale Data Archiving and Processing Section, Institute of Economic Research, Hitotsubashi University, Tokyo, Japan; 4 AMR Clinical Reference Center, National Center for Global Health and Medicine, Tokyo, Japan

**Keywords:** Coronavirus disease 2019 (COVID-19), Disseminated intravascular coagulation (DIC), Organ dysfunction, Incidence, Prognosis, Diseases, Haematological diseases, Infectious diseases

## Abstract

This study aimed to investigate the incidence and significance of disseminated intravascular coagulation (DIC) in coronavirus disease 2019 (COVID-19). A multicenter cohort study was conducted using large-scale COVID-19 registry data. The patients were classified into DIC and non-DIC groups based on the diagnosis on admission (day 1) and on any of the days 1, 4, 8, and 15. In total, 23,054 patients were divided into DIC (n = 264) and non-DIC (n = 22,790) groups on admission. Thereafter, 1654 patients were divided into 181 patients with DIC and 1473 non-DIC patients based on the DIC diagnosis on any of the days from 1 to 15. DIC incidence was 1.1% on admission, increasing to 10.9% by day 15. DIC diagnosis on admission had moderate predictive performance for developing multiple organ dysfunction syndrome (MODS) on day 4 and in-hospital death and was independently associated with MODS and in-hospital death. DIC diagnosis on any of the days from 1 to 15, especially days 8 and 15, was associated with lower survival probability than those without DIC and showed significant association with in-hospital death. In conclusion, despite its low incidence, DIC, particularly late-onset DIC, plays a significant role in the pathogenesis of poor prognosis in patients with COVID-19.

## Introduction

The development of serious coagulopathy is acknowledged in coronavirus disease 2019 (COVID-19). The main pathological mechanisms of coagulopathy include excess immune and inflammatory responses, platelet hyperactivation, hypercoagulability, and endothelial injury associated with an insufficient anticoagulation system^1^. These changes lead to arterial and venous thrombus formation and microvascular thrombosis, resulting in multiple organ dysfunction syndrome (MODS) and poor prognosis for patients with COVID-19^1^. COVID-19 also affects the fibrinolytic system^2^. While increased plasminogen activator inhibitor-1 accelerates thrombus formation, expressions of genes encoding tissue- and urokinase-type plasminogen activator may induce bleeding complications^3,4^. Initially attributed to disseminated intravascular coagulation (DIC) during the early stage of the COVID-19 pandemic, these changes did not fully meet the International Society on Thrombosis and Haemostasis (ISTH) DIC diagnostic criteria due to small changes in platelet counts and coagulofibrinolytic parameters except D-dimer^5,6^. The main pathophysiology of COVID-19-associated coagulopathy is distinct from DIC but partially overlaps with DIC, thrombotic microangiopathy, cytokine release syndrome, and catastrophic antiphospholipid syndrome^7,8^.

DIC is a frequent complication of sepsis and is associated with MODS, often leading to poor prognosis^9^. COVID-19 has been considered a severe acute respiratory syndrome coronavirus 2 (SARS-CoV-2)-induced viral sepsis^10^. Most patients with COVID-19 met the definition of Sepsis-3 criteria, developed MODS requiring ICU admission and artificial organ support, and were associated with high ICU mortality^10,11^. These patients also experienced coagulopathy defined by Sepsis-3 using the sequential organ failure assessment (SOFA) score^11^. While an early report on DIC in COVID-19 showed a high incidence of DIC (71.4%) in non-survivors, later systematic reviews and meta-analyses confirmed a low incidence of DIC from 3.0 to 4.3%^12–14^. Despite its low incidence, DIC correlated with COVID-19 severity and poor prognosis. Additionally, deaths were more likely to occur in patients with DIC^14^. These meta-analyses indicate the important role of DIC in SARS-CoV-2-induced viral sepsis; however, the results were limited by the heterogeneity of cited studies and diverse criteria and timing of DIC diagnosis. Furthermore, included patients were restricted to the very early stage of the first several months from the onset of the COVID-19 pandemic.

This study aimed to investigate the incidence of DIC in COVID-19 using large-scale registry data and test the hypothesis that DIC is independently associated with poor prognosis of patients with COVID-19.

## Materials and methods

### Study design, setting, and ethical approval

This is a multicenter retrospective cohort study using prospectively collected nationwide registry data from the COVID-19 Registry Japan (COVIREGI-JP), operated under the Repository of Data and Biospecimen of Infectious Disease (REBIND) project commissioned by the Ministry of Health, Labour and Welfare of Japan. In August 2023, 677 hospitals participated in COVIREGI-JP. Patient data were registered at the Research Electronic Data Capture, a web-based data capture application hosted at the Joint Center for Researchers, Associates, and Clinicians data center of the National Center for Global Health and Medicine. The data used were provided by COVIREGI-JP, and the study project was approved by the Ethics Committee of the research institute (TEG-01791-012) after a document on an opt-out policy for all subjects and/or their relatives was uploaded on the website of the institute. Written informed consent was waived because the data collection was performed without any interventions, and data management was processed anonymously. All research procedures were performed in accordance with the Declaration of Helsinki.

### Participants

**Figure 1:**
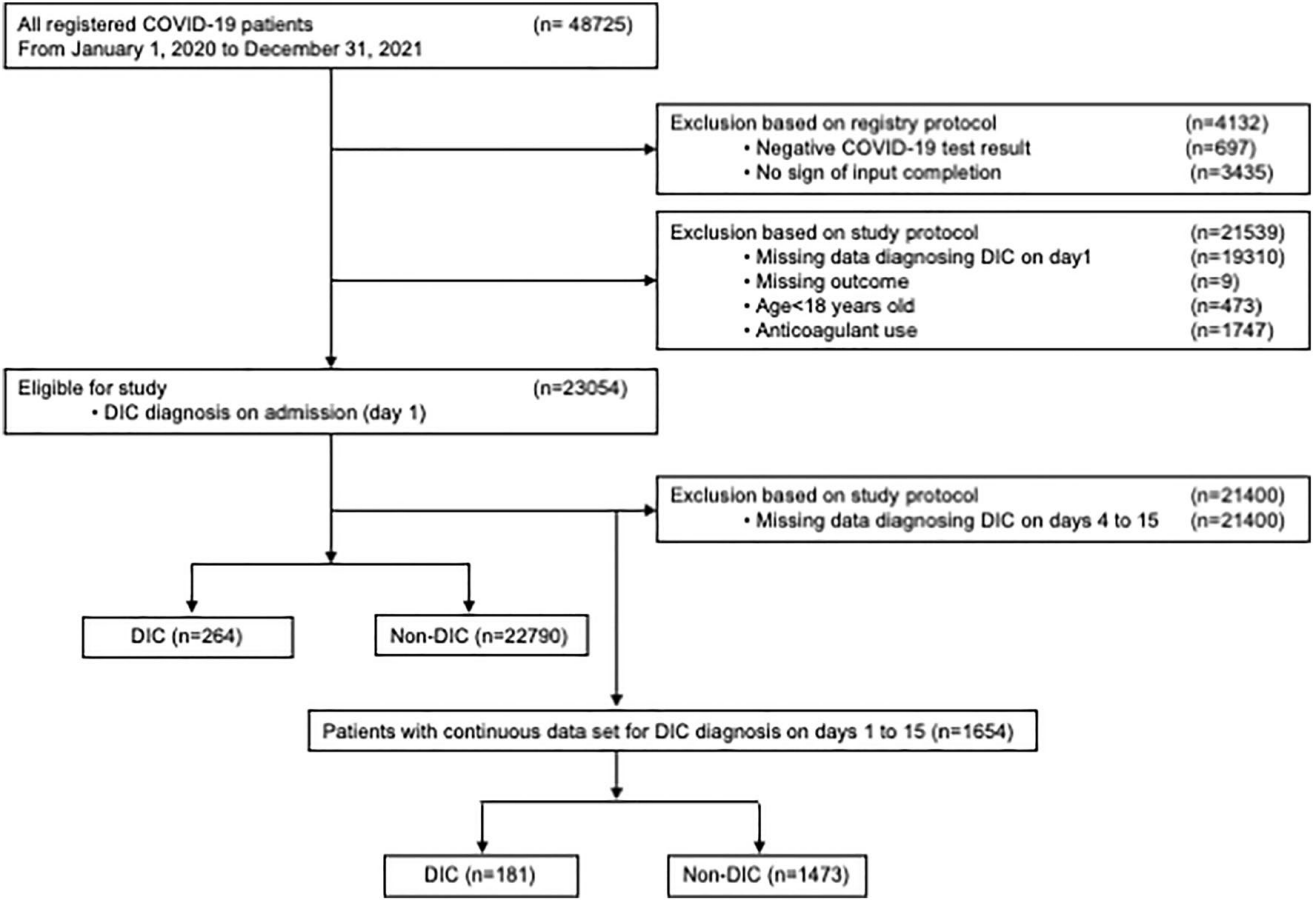
Flow chart of the inclusion and exclusion of the study.

Eligible participants included all adult patients (age ≥ 18 years) diagnosed with COVID-19 by SARS-CoV-2 rapid antigen test or polymerase chain reaction test and admitted to the hospital. Patients using anticoagulants and those lacking data for DIC diagnosis on admission (day 1) were excluded. In the first analysis, the included patients were divided into DIC and non-DIC groups for analysis on admission. In the second analysis, patients without continuous data for diagnosing DIC from days 1 to 15 were further excluded. Those with complete continuous data for DIC diagnosis were then divided into DIC and non-DIC groups based on the DIC diagnosis on any of the days from 1 to 15. The enrollment period of the present study was from January 1, 2020, to December 31, 2021. Flow chart of the inclusion and exclusion of patients is shown in Fig. 1.

### Diagnosis and definition

A DIC diagnosis was made based on the Japanese Association for Acute Medicine (JAAM) DIC diagnostic criteria^15^ (Supplementary Fig. 1). In this study, the prothrombin time/international normalized ratio (INR) served as a substitute for the prothrombin time ratio in DIC diagnosis. In cases where fibrinogen/fibrin degradation products (FDP) data were missing, D-dimer levels were used as a substitute. The systemic inflammatory response syndrome (SIRS) and quick SOFA (qSOFA) were calculated using the definitions of the American College of Chest Physicians/Society of Critical Care Medicine and Sepsis-3, respectively^11,16^. Berlin definition was used for acute respiratory distress syndrome (ARDS) diagnosis and severity classification^17^. The severity of hypoxemia of the patients was defined as follows: mild, SpO_2_ ≥ 94% under room air; moderate, SpO_2_ < 94% under room air or need for oxygen at any flow rate including nasal high flow oxygenation; and severe, use of invasive or non-invasive ventilation or extracorporeal membrane oxygenation (ECMO). Shock was defined as mean arterial pressure less than 65 mmHg on admission. Organ dysfunction was diagnosed on day 4, and the definitions were as follows: central nervous system, alert, respond to voice, respond to pain, unresponsive (AVPU) scale ≥ 1^18^; lung, use any one of the following treatments (oxygen, nasal high flow oxygen, invasive and non-invasive ventilation, prone ventilation, nitric oxide, tracheostomy, muscle relaxant, extracorporeal membrane oxygenation [ECMO]); heart, use of catecholamines or inotropes; liver, total bilirubin ≥ 2.0 mg/dL; and kidney, use of renal replacement therapy or peritoneal dialysis. More than two organ dysfunctions were defined as MODS. Body mass index (BMI) was calculated, and obesity levels were defined as follows^19^: underweight, < 18.5; normal, 18.5–24.9; obesity 1, 25.0–29.9; obesity 2, 30.0–39.9; and obesity 3, ≥ 40 kg/m^2^. Comorbidities and complications were diagnosed at the discretion of the physicians of participating institutes.

### Data collection and measurements

Laboratory data on clinical signs and treatments were registered on admission (day 1) and on days 4, 8, and 15. Apart from the routine laboratory tests, platelet counts, prothrombin time (INR), activated partial thromboplastin time (APTT), fibrinogen, FDP, and D-dimer were measured for evaluating coagulofibrinolytic changes and DIC diagnosis.

### Outcome measures

Firstly, this study determined the incidence of DIC in COVID-19 in the two cohorts. The first cohort comprised patients with COVID-19 diagnosed with DIC on admission (day 1), and the second cohort included patients with complete DIC diagnostic data from day 1 to days 4, 8, and 15 after admission. In the second cohort, patients diagnosed with DIC on any of the days from 1 to 15 were classified into the DIC group. The primary outcome was in-hospital death, and secondary outcomes included the number of organ dysfunction, incidence of MODS, ICU admission, and hospital length of stay. These outcomes were compared between patients with DIC and non-DIC patients while evaluating the corresponding influences of DIC. Potential confounders for evaluating in-hospital death were age, sex, BMI, comorbidities, shock, oxygen saturation (SpO_2_), lymphocyte, and complications. Upon assessing MODS on day 4, age, sex, BMI, comorbidities, shock, oxygen saturation, and lymphocyte were used as potential confounders. All patients were followed-up until hospital discharge.

### Statistical analyses

Measured variables were expressed as the median with the 25–75 interquartile range or number in percentage. Study size depended on the registration period, and missing values were used without imputation. Differences in demographics and measured parameters between the two groups (DIC vs. non-DIC) were compared using the Mann–Whitney U-test and Fisher’s exact test for continuous and nominal variables, respectively. The receiver operating characteristic (ROC) curve was constructed, and the area under the ROC curve (AUC) was used to assess the predictive ability of DIC scores for MODS and in-hospital death. Youden’s index was applied in computing cut-off values for sensitivity and specificity. Survival probability curves of patients with and without DIC were derived based on the Kaplan–Meier method. Univariate and multivariate logistic regression models were applied to obtain crude and adjusted odds ratios (OR), respectively, and 95% confidence intervals (CI) were used to confirm the independent association between DIC with in-hospital death and MODS on day 4. Sensitivity analyses were applied with stepwise logistic regression analyses using the Akaike information criterion in the backward elimination method using similar potential confounders described in the section on outcome measures. The Wald test was used to evaluate the significance of the results from the logistic regression analyses. The Hosmer–Lemeshow goodness-of-fit test, variance inflation factor (VIF), and Cook’s distances were used as diagnostic statistics to evaluate the model fit, collinearity, and influential points, respectively, to confirm the robustness of the logistic regression analyses.

Differences with a two-tailed *p*-value of < 0.05 were considered statistically significant. The R (version 4.2.3; A language and environment for statistical computing. R foundation for statistical computing, Vienna, Austria) was used for the statistical analyses and calculations.

## Results

### Characteristics of the patients

In total, 48,725 patients were included during the study period. After the eligibility assessment, 23,054 patients were included in the final analysis (Fig. 1). Enrolled patients were divided into 264 patients with DIC and 22,790 non-DIC patients based on the DIC diagnosis on admission. From the enrolled 23,054 patients, 21,400 patients with missing data for continuous DIC diagnosis from day 1 to days 4, 8, and 15 were excluded. In total, 1654 patients were eligible for the second analysis and were divided into 181 patients with DIC and 1473 non-DIC patients based on the DIC diagnosis on any of the days from 1 to 15. Tables 1 and 2 present the characteristics of the 23,054 patients and 1654 patients for the primary and secondary analyses, respectively.

**Table 1:** Characteristics of patients with a possible of DIC diagnosis on admission.

	All patients (n = 23,054)	Non-DIC (n = 22,790)	DIC (n = 264)	*p*-value
Age (years)	61.0 [45.0, 76.0]	61.0 [45.0, 76.0]	73.0 [64.8, 82.0]	< 0.001
Male sex, n (%)	13,500 (58.6)	13,318 (58.4)	182 (68.9)	< 0.001
Weight (kg)	63.7 [53.1, 75.0]	63.8 [53.1, 75.0]	60.0 [52.2, 71.5]	0.010
Hight (cm)	164.0 [156.0, 170.0]	164.0 [156.0, 170.0]	163.0 [156.0, 170.0]	0.599
Body mass index, n (%)	23.8 [21.2, 26.8]	23.8 [21.2, 26.9]	22.9 [20.3, 25.3]	0.002
Comorbidity, n (%)	14,106 (61.2)	13,887 (61.0)	219 (83.0)	< 0.001
Comorbidity, n	1.0 [0.0, 2.0]	1.0 [0.0, 2.0]	2.0 [1.0, 3.0]	< 0.001
**Medications**				
Angiotensin converting enzyme inhibitors, n (%)	427 (1.9)	421 (1.8)	6 (2.3)	0.495
Angiotensin II receptor antagonists, n (%)	3812 (16.5)	3768 (16.5)	44 (16.7)	0.934
Antiplatelet drugs, n (%)	1892 (8.2)	1860 (8.2)	32 (12.1)	0.024
Anticoagulants during stay, n (%)	6351 (27.6)	6212 (27.3)	139 (52.9)	< 0.001
**Severity of illness**				
DIC score	0.0 [0.0, 0.0]	0.0 [0.0, 0.0]	4.0 [4.0, 5.0]	< 0.001
Shock, n (%)	235 (1.0)	221 (1.0)	14 (5.3)	< 0.001
SIRS criteria	1.0 [1.0, 2.0]	1.0 [1.0, 2.0]	3.0 [2.0, 3.0]	< 0.001
SIRS, n (%)	8346 (36.2)	8138 (35.7)	208 (78.8)	< 0.001
qSOFA criteria	0.0 [0.0, 1.0]	0.0 [0.0, 1.0]	1.0 [0.0, 1.0]	< 0.001
qSOFA, n (%)	795 (3.6)	737 (3.4)	58 (24.2)	< 0.001
Oxygen saturation (%)	97.0 [95.0, 98.0]	97.0 [95.0, 98.0]	95.0 [92.0, 97.0]	< 0.001
Severity of hypoxemia				
Mild	15,838 (73.0)	15,783 (73.6)	55 (22.0)	
Moderate	5151 (23.7)	5001 (23.3)	150 (60.0)	
Severe	706 (3.3)	661 (3.1)	45 (18.0)	< 0.001
**Clinical signs**				
Glasgow Coma Scale	15.0 [15.0, 15.0]	15.0 [15.0, 15.0]	15.0 [12.0, 15.0]	< 0.001
AVPU scale				
Alert	21,557 (95.4)	21,370 (95.7)	187 (73.9)	
Verbal	792 (3.5)	750 (3.4)	42 (16.6)	
Pain	160 (0.7)	149 (0.7)	11 (4.3)	
Unresponsive	81 (0.4)	68 (0.3)	13 (5.1)	< 0.001
Temperature (℃)	37.0 [36.6, 37.7]	37.0 [36.6, 37.7]	37.2 [36.6, 38.2]	0.004
Heart rate (beats/min)	86.0 [75.0, 97.0]	86.0 [75.0, 97.0]	95.5 [81.0, 110.0]	< 0.001
Respiratory rate (breaths/min)	18.0 [16.0, 21.0]	18.0 [16.0, 21.0]	22.0 [18.0, 26.0]	< 0.001
Systolic blood pressure (mmHg)	129.0 [116.0, 144.0]	129.0 [116.0, 143.0]	130.0 [114.0, 146.2]	0.577
Diastolic blood pressure (mmHg)	79.0 [70.0, 89.0]	79.0 [70.0, 89.0]	77.0 [66.0, 87.0]	0.001
Mean blood pressure (mmHg)	96.0 [86.7, 106.0]	96.0 [86.7, 106.0]	94.7 [82.9, 105.3]	0.073
**Laboratory findings**				
White blood cell count (10^3^/µL)	5.2 [4.0, 6.8]	5.1 [4.0, 6.7]	7.7 [3.9, 12.7]	< 0.001
Lymphocyte (%)	20.9 [13.4, 29.0]	21.0 [13.5, 29.1]	9.2 [5.2, 18.0]	< 0.001
Hemoglobin (g/dL)	14.0 [12.7, 15.2]	14.0 [12.7, 15.3]	12.6 [10.7, 14.0]	< 0.001
Hematocrit (%)	41.2 [37.6, 44.5]	41.2 [37.6, 44.5]	36.7 [31.8, 41.4]	< 0.001
Platelet count (10^3^/µL)	191.0 [153.0, 239.0]	192.0 [153.0, 239.0]	106.0 [73.0, 207.5]	< 0.001
Activated partial thromboplastin time (sec)	32.1 [29.3, 35.4]	32.0 [29.3, 35.4]	33.7 [29.8, 39.3]	< 0.001
Prothrombin time international normalized ratio	1.0 [1.0, 1.1]	1.0 [1.0, 1.1]	1.2 [1.1, 1.3]	< 0.001
Fibrinogen (mg/dL)	440.0 [343.0, 554.0]	440.9 [343.0, 554.5]	406.9 [286.0, 532.0]	< 0.001
FDP (µg/mL)	3.0 [2.5, 4.7]	3.0 [2.5, 4.5]	37.7 [16.1, 98.8]	< 0.001
D-dimer (ng/mL)	800.0 [500.0, 1460.0]	800.0 [500.0, 1400.0]	17,850.0 [1917.5, 53,425.0]	< 0.001
Albumin (g/dL)	3.8 [3.3, 4.2]	3.8 [3.3, 4.2]	2.8 [2.3, 3.3]	< 0.001
Total bilirubin (mg/dL)	0.6 [0.4, 0.7]	0.6 [0.4, 0.7]	0.8 [0.5, 1.1]	< 0.001
AST (U/L)	30.0 [22.0, 45.0]	30.0 [22.0, 45.0]	46.0 [29.8, 67.8]	< 0.001
ALT (U/L)	24.0 [16.0, 41.0]	24.0 [16.0, 41.0]	28.0 [18.0, 45.0]	0.018
CRP mg/dL)	2.4 [0.5, 7.0]	2.3 [0.4, 6.9]	10.0 [3.8, 18.4]	< 0.001
BUN (mg/dL)	14.0 [10.8, 19.0]	14.0 [10.8, 18.9]	24.4 [15.6, 36.0]	< 0.001
Creatinine (mg/dL)	0.8 [0.7, 1.0]	0.8 [0.7, 1.0]	0.9 [0.7, 1.4]	< 0.001
Lactate (mmol/L)	1.3 [1.0, 1.7]	1.3 [1.0, 1.7]	1.8 [1.2, 2.9]	< 0.001
**Complications and outcomes**				
Complications, n (%)	2977 (13.0)	2865 (12.6)	112 (43.1)	< 0.001
Complications, n	0.0 [0.0, 0.0]	0.0 [0.0, 0.0]	0.0 [0.0, 1.0]	< 0.001
Hospital length of stay (days)	10.0 [7.0, 15.0]	10.0 [7.0, 15.0]	15.0 [7.0, 24.0]	< 0.001
Intensive care unit admission, n (%)	2657 (11.5)	2544 (11.2)	113 (42.8)	< 0.001
Number of organ dysfunction	1.0 [0.0, 1.0]	1.0 [0.0, 1.0]	1.0 [1.0, 2.0]	< 0.001
Multiple organ dysfunction syndrome, n (%)	1319 (10.7)	1250 (10.3)	69 (41.3)	< 0.001
In-hospital death, n (%)	1326 (5.8)	1249 (5.5)	77 (29.2)	< 0.001

*AVPU* alert, verbal, pain, and unresponsive, *DIC* disseminated intravascular coagulation, *FDP* fibrin/fibrinogen degradation products, *n* number, *qSOFA* quick sequential organ failure assessment, *SIRS* systemic inflammatory response syndrome. All data except complications and outcomes were obtained at admission (Day 1).

**Table 2:** Characteristics of patients with a possible DIC diagnosis on any of the days 1, 4, 8, and 15.

	All patients (n = 1654)	Non-DIC (n = 1473)	DIC (n = 181)	*p*-value
Age (years)	71.0 [61.0, 80.0]	71.0 [60.0, 80.0]	72.0 [65.0, 79.0]	0.302
Male sex n (%)	1091 (66.0)	956 (64.9)	135 (74.6)	0.001
Weight (kg)	64.8 [54.0, 75.0]	64.8 [54.0, 75.0]	65.0 [53.0, 73.3]	0.479
Hight (cm)	164.0 [155.1, 170.0]	164.0 [155.0, 170.0]	163.0 [157.0, 170.0]	0.990
Body mass index n (%)	24.2 [21.7, 27.2]	24.3 [21.8, 27.4]	23.8 [21.3, 26.2]	0.098
Comorbidity, n (%)	1352 (81.7)	1191 (80.9)	161 (89.0)	0.008
Comorbidity, n	2.0 [1.0, 3.0]	2.0 [1.0, 3.0]	2.0 [1.0, 3.0]	0.073
**Medications**				
Angiotensin converting enzyme inhibitors, n (%)	47 (2.8)	44 (3.0)	3 (1.7)	0.474
Angiotensin II receptor antagonists, n (%)	406 (24.5)	364 (24.7)	42 (23.2)	0.715
Antiplatelet drugs, n (%)	231 (14.0)	197 (13.4)	34 (18.8)	0.053
Anticoagulants during stay, n (%)	1135 (68.7)	1000 (68.0)	135 (74.6)	0.075
**Severity of illness**				
DIC				
Day 1				
DIC score	0.0 [0.0, 1.0]	0.0 [0.0, 1.0]	2.0 [1.0, 4.0]	< 0.001
DIC yes n (%)	63 (3.8)	0 (0.0)	63 (34.8)	< 0.001
Day 4				
DIC score	0.0 [0.0, 1.0]	0.0 [0.0, 1.0]	2.0 [1.0, 4.0]	< 0.001
DIC yes n (%)	58 (3.5)	0 (0.0)	58 (32.0)	< 0.001
Day 8				
DIC score	0.0 [0.0, 1.0]	0.0 [0.0, 1.0]	2.0 [1.0, 4.0]	< 0.001
DIC yes n (%)	59 (3.6)	0 (0.0)	59 (32.6)	< 0.001
Day 15				
DIC score	0.0 [0.0, 1.0]	0.0 [0.0, 1.0]	2.0 [1.0, 4.0]	< 0.001
DIC yes n (%)	73 (4.4)	0 (0.0)	73 (40.3)	< 0.001
Shock, n (%)	30 (1.8)	24 (1.6)	6 (3.3)	0.132
SIRS criteria	2.0 [1.0, 2.0]	2.0 [1.0, 2.0]	2.0 [1.0, 3.0]	< 0.001
SIRS, n (%)	873 (52.8)	756 (51.3)	117 (64.6)	0.001
qSOFA criteria	0.0 [0.0, 1.0]	0.0 [0.0, 1.0]	1.0 [0.0, 1.0]	< 0.001
qSOFA, n (%)	137 (8.9)	101 (7.3)	36 (21.7)	< 0.001
Oxygen saturation (%)	95.0 [93.0, 97.0]	96.0 [93.0, 97.0]	95.0 [91.0, 97.0]	0.001
Severity of hypoxemia				
Mild	639 (41.3)	614 (44.7)	25 (14.4)	
Moderate	696 (45.0)	601 (43.7)	95 (54.6)	
Severe	213 (13.8)	159 (11.6)	54 (31.0)	< 0.001
**Clinical signs**				
Glasgow Coma Scale	15.0 [15.0, 15.0]	15.0 [15.0, 15.0]	15.0 [10.0, 15.0]	< 0.001
AVPU criteria				
Alert	1413 (88.8)	1289 (90.6)	124 (73.4)	
Verbal	123 (7.7)	92 (6.5)	31 (18.3)	
Pain	42 (2.6)	32 (2.2)	10 (5.9)	
Unresponsive	14 (0.9)	10 (0.7)	4 (2.4)	< 0.001
Temperature (℃)	37.3 [36.7, 38.1]	37.3 [36.7, 38.1]	37.1 [36.6, 37.9]	0.013
Heart rate (beats/min)	88.0 [76.0, 100.0]	88.0 [76.0, 100.0]	89.0 [76.0, 103.0]	0.126
Respiratory rate (breaths/min)	20.0 [18.0, 24.0]	20.0 [18.0, 24.0]	22.0 [18.0, 26.0]	0.002
Systolic blood pressure (mmHg)	132.0 [118.0, 147.0]	132.0 [119.0, 147.0]	129.0 [116.0, 149.0]	0.237
Diastolic blood pressure (mmHg)	78.0 [69.0, 88.0]	78.0 [69.0, 88.0]	74.0 [64.0, 83.0]	< 0.001
Mean blood pressure (mmHg)	96.0 [86.7, 105.7]	96.3 [87.3, 106.0]	92.7 [82.3, 104.3]	0.003
Laboratory findings on day1				
White blood cell count (10^3^/µL)	5.8 [4.3, 8.5]	5.7 [4.2, 8.1]	7.5 [5.0, 11.5]	< 0.001
Lymphocyte (%)	14.0 [7.9, 21.9]	14.9 [8.4, 22.8]	8.8 [5.0, 14.9]	< 0.001
Hemoglobin (g/dL)	13.6 [12.2, 14.9]	13.7 [12.3, 15.0]	12.9 [11.2, 14.4]	< 0.001
Hematocrit (%)	40.1 [36.0, 43.6]	40.3 [36.3, 43.8]	38.4 [33.7, 41.7]	< 0.001
Albumin (g/dL)	3.3 [2.9, 3.7]	3.4 [3.0, 3.8]	2.9 [2.5, 3.2]	< 0.001
Total bilirubin (mg/dL)	0.6 [0.4, 0.8]	0.6 [0.4, 0.7]	0.7 [0.4, 0.9]	< 0.001
AST (U/L)	39.0 [27.0, 58.0]	38.0 [27.0, 56.0]	47.0 [31.8, 72.2]	< 0.001
ALT (U/L)	27.0 [17.0, 45.0]	26.0 [17.0, 44.2]	29.0 [17.0, 48.0]	0.425
CRP mg/dL)	6.4 [2.5, 11.9]	5.9 [2.2, 11.1]	10.5 [6.3, 16.6]	< 0.001
BUN (mg/dL)	17.9 [13.1, 25.0]	17.0 [13.0, 24.0]	22.9 [16.1, 33.6]	< 0.001
Creatinine (mg/dL)	0.9 [0.7, 1.1]	0.9 [0.7, 1.1]	0.9 [0.7, 1.4]	0.004
Lactate (mmol/L)	1.3 [1.0, 1.8]	1.3 [1.0, 1.7]	1.4 [1.0, 2.0]	0.137
**Complications and outcomes**				
Complications, n (%)	721 (43.9)	582 (39.7)	139 (77.7)	< 0.001
Complications, n	0.0 [0.0, 1.0]	0.0 [0.0, 1.0]	1.5 [1.0, 2.0]	< 0.001
Hospital length of stay (days)	22.0 [17.0, 33.0]	22.0 [17.0, 31.0]	26.0 [19.0, 41.0]	< 0.001
Intensive care unit admission, n (%)	691 (41.8)	553 (37.6)	138 (76.2)	< 0.001
Number of organ dysfunction	1.0 [1.0, 2.0]	1.0 [1.0, 1.0]	2.0 [1.0, 3.0]	< 0.001
Multiple organ dysfunction syndrome, n (%)	408 (28.5)	308 (24.0)	100 (66.2)	< 0.001
In-hospital death, n (%)	263 (15.9)	182 (12.4)	81 (44.8)	< 0.001

*AVPU* alert, verbal, pain, and unresponsive, *DIC* disseminated intravascular coagulation, *FDP* fibrin/fibrinogen degradation products, *n* number, *qSOFA* quick sequential organ failure assessment, *SIRS* systemic inflammatory response syndrome.

### Incidence of DIC, bleeding, and thrombotic complications

The incidence of DIC on admission was 1.1% (264/23,054), which increased to 10.9% (181/1,654) when it could be diagnosed on any of the days 1, 4, 8, and 15 after admission. The percentage of DIC diagnoses among the 1,654 patients was as follows: day 1 (34.8%), day 4 (32.0%), day 8 (32.6%), and day 15 (40.3%), as shown in Table 2. Patients with DIC exhibited higher severity of illness and worse clinical signs and laboratory findings than non-DIC patients. Additionally, these patients presented with more comorbidities and complications. Concrete diagnoses of comorbidities and complications are presented in Supplementary Tables 1 and 2. The patients with DIC, presented in these Supplementary Tables, showed significantly higher incidences of gastrointestinal bleeding, deep vein thrombosis, and pulmonary thromboembolism on admission.

### Outcomes of the patients

Longer hospital length of stay, higher percentage of ICU admission, greater organ dysfunction, and higher incidence of MODS were observed in patients with DIC than non-DIC patients, contributing to higher in-hospital death rate (DIC vs. non-DIC, Table 1: 29.2 vs. 5.5%, *p* < 0.001; Table 2: 44.8 vs. 12.4%, *p* < 0.001) (Tables 1 and 2).

### Survival probability and outcome prediction

**Figure 2:**
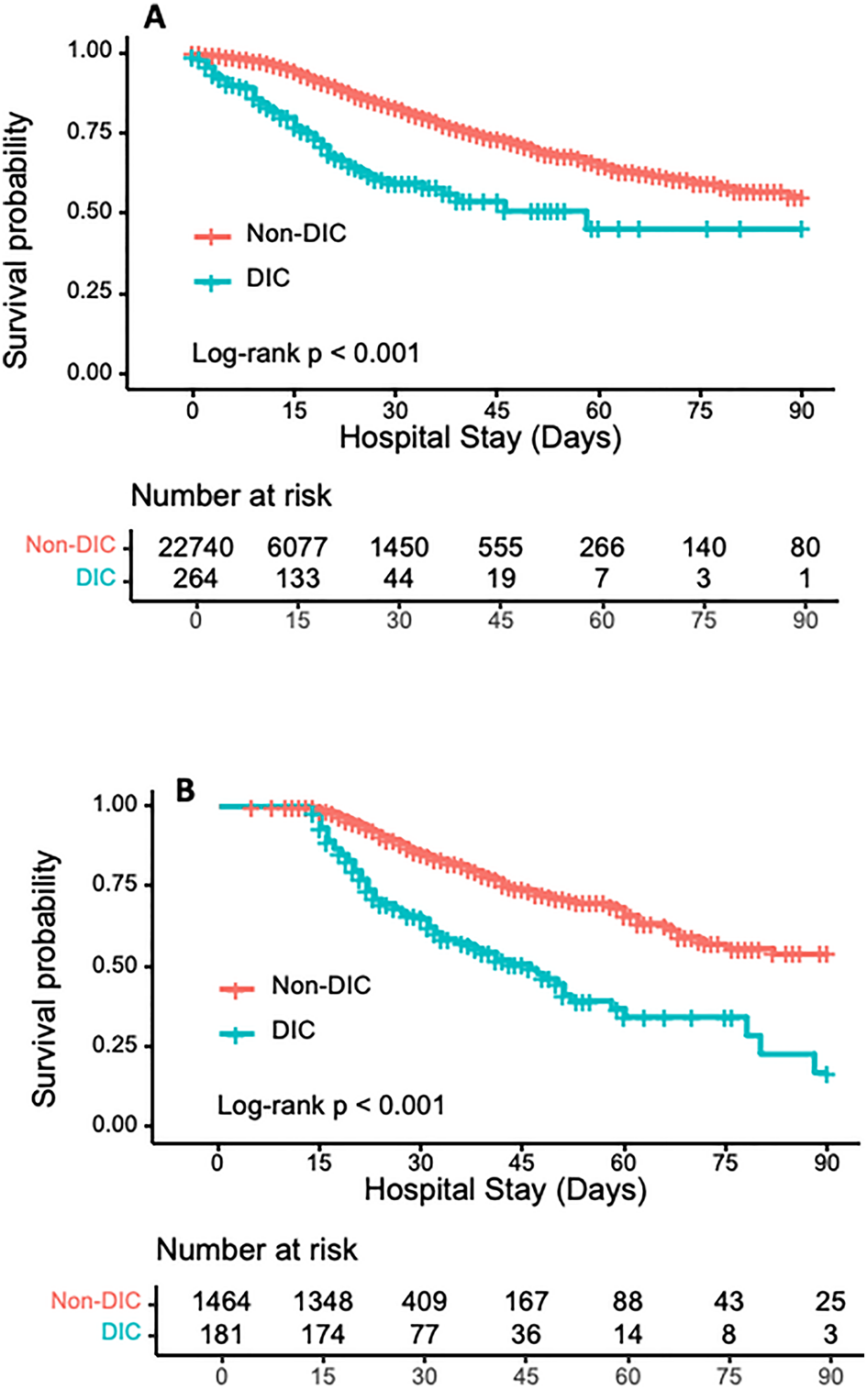
Kaplan–Meier survival probability curves from enrollment to day 90. (**A**) patients diagnosed with DIC on admission; (**B**) patients diagnosed with DIC on any of days 1, 4, 8, and 15. Both (**A**) and (**B**) show significantly lower survival probability of patients with DIC than those without DIC. The numbers of graphs represent the number of DIC and non-DIC patients at risk of death on the indicated days. DIC, disseminated intravascular coagulation.

**Figure 3:**
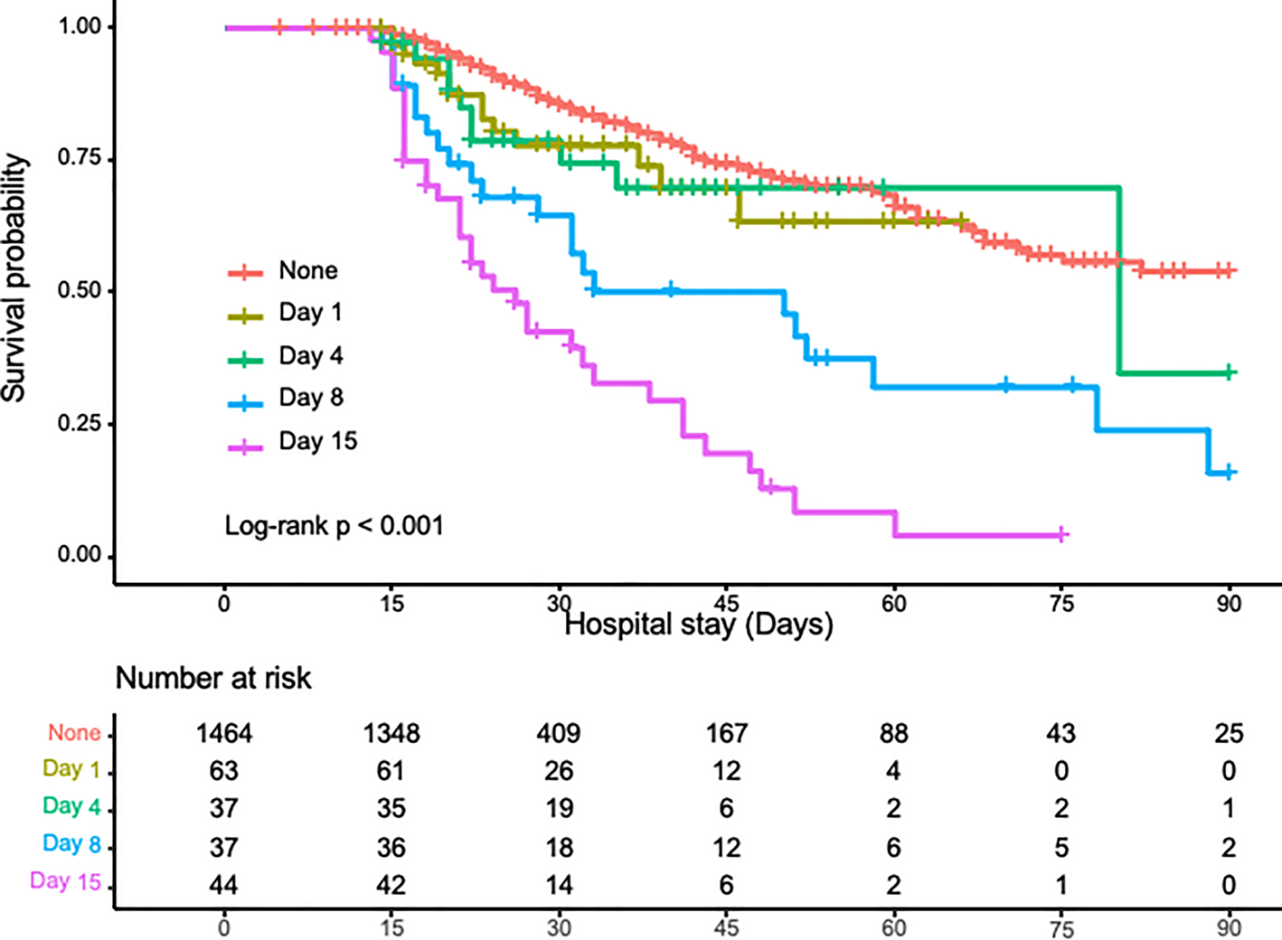
Kaplan–Meier survival probability curves from enrollment to day 90 of patients diagnosed with DIC on any of the days 1, 4, 8, and 15 and non-DIC patients. Significant differences in the survival probabilities of patients were found. Survival probabilities of patients diagnosed with DIC on day 8 were significantly lower than non-DIC patients (*p* < 0.001) and DIC patients diagnosed on day 4 (*p* < 0.0351). Additionally, significant differences in survival probabilities between day 15 and non-DIC (*p* < 0.001), day 15 and day 1 (*p* < 0.001), day 15 and day 4 (*p* < 0.001), and day 15 and day 8 (*p* < 0.0027) were observed. These suggest a lower survival probability in late-onset DIC than in early-onset DIC. The numbers of the graphs represent the number of DIC each day and non-DIC patients at risk of death on the indicated days. DIC, disseminated intravascular coagulation.

The survival probability of patients with DIC diagnosed on admission was lower than non-DIC patients (*p* < 0.001), as shown in Fig. 2A. Figure 2B revealed a lower survival probability of patients with DIC diagnosed during the observation period from days 1 to 15 than non-DIC patients (*p* < 0.001). Furthermore, survival probabilities were significantly lower in late-onset DIC diagnosed on day 8 and day 15 than in non-DIC and early-onset DIC (day 8 vs. non-DIC, *p* < 0.001; day 8 vs. day 4, *p* < 0.0351) and (day 15 vs. non-DIC, *p* < 0.001; day 15 vs. day 1, *p* < 0.001; day 15 vs. day 4, *p* < 0.001; day 15 vs. day 8, *p* < 0.0027). Results are presented in Fig. 3.

**Figure 4:**
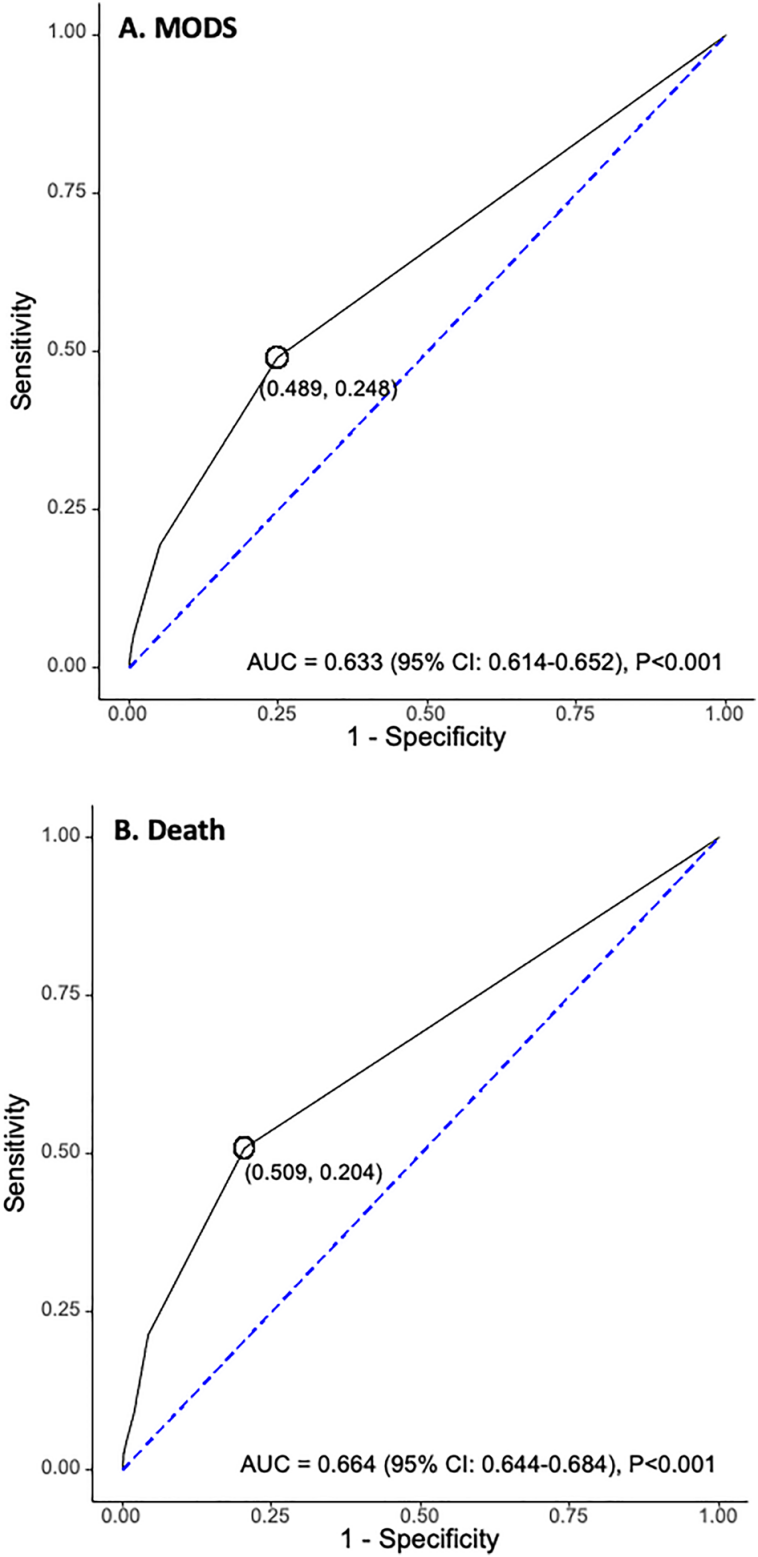
Predictive performance of DIC diagnosed on admission for developing MODS on day 4 (**A**) and in-hospital death (**B**). The ROC curve analyses showed moderate but significant predictive performance of DIC diagnosis for MODS (sensitivity 48.9 and specificity 75.2%) and in-hospital death (sensitivity 50.9% and specificity 79.6%). AUC, area under ROC curve; MODS, multiple organ dysfunction syndrome; ROC, receiver operating characteristic.

The ROC curve analyses showed moderate predictive ability of DIC scores on day 1 for MODS on day 4 (AUC 0.633, *p* < 0.001) and in-hospital death (AUC 0.664, *p* < 0.001) of patients with COVID-19 (Fig. 4). Multivariable logistic regression analyses demonstrated that DIC diagnosis on admission was independently associated with MODS on day 4 (OR 3.12, 95% CI 2.07–4.68, *p* < 0.001) and in-hospital death (OR 2.03, 95% CI 1.33–3.11, *p* = 0.001) of patients with COVID-19 (Table 3). DIC diagnosis during the observation period from days 1 to 15 was also shown to be an independent predictor of these patients (OR 4.76, 95% CI 3.07–7.39, *p* < 0.001) (Table 4). Sensitivity analyses by stepwise logistic regression analysis confirmed similar results, indicating that DIC diagnosis on admission and during the observation period of the first two weeks was independently associated with in-hospital death and MODS on day 4 and in-hospital death, respectively (Supplementary Tables 3 and 4).

**Table 3:** Logistic regression analysis for in-hospital death and MODS on day 4 in patients with a possible DIC diagnosis on admission.

Parameters	Crude OR (95%CI)	*p*-value	Adjusted OR (95% CI)	*p*-value
**In-hospital death**				
DIC	7.56 (5.39, 10.61)	< 0.001	2.03 (1.33, 3.11)	0.001
Age (years)	1.09 (1.08, 1.09)	< 0.001	1.10 (1.09, 1.10)	< 0.001
Male sex	1.19 (1.03, 1.37)	0.017	1.61 (1.35, 1.91)	< 0.001
Body mass index				
Underweight	1.90 (1.54, 2.34)	< 0.001	1.14 (0.89, 1.46)	0.290
Obesity 1	0.90 (0.76, 1.06)	0.224	1.15 (0.94, 1.39)	0.170
Obesity 2	0.64 (0.48, 0.85)	0.002	1.13 (0.80, 1.60)	0.478
Obesity 3	0.35 (0.11, 1.10)	0.072	0.76 (0.19, 3.03)	0.700
Comorbidities (n)	1.71 (1.64, 1.79)	< 0.001	1.21 (1.14, 1.29)	< 0.001
Shock	2.95 (1.86, 4.67)	< 0.001	1.71 (0.98, 2.96)	0.057
Oxygen saturation (%)	0.87 (0.86, 0.89)	< 0.001	0.95 (0.94, 0.97)	< 0.001
Lymphocyte (%)	0.91 (0.91, 0.92)	< 0.001	0.96 (0.96, 0.97)	< 0.001
Complications (n)	4.30 (3.97, 4.66)	< 0.001	3.07 (2.81, 3.36)	< 0.001
**MODS on day 4**				
DIC	7.21 (5.02, 10.36)	< 0.001	3.12 (2.07, 4.68)	< 0.001
Age (years)	1.04 (1.03, 1.04)	< 0.001	1.03 (1.02, 1.03)	< 0.001
Male sex	1.41 (1.23, 1.63)	< 0.001	1.49 (1.27, 1.74)	< 0.001
Body mass index				
Underweight	1.67 (1.33, 2.09)	< 0.001	1.39 (1.08, 1.79)	0.010
Obesity 1	0.91 (0.77, 1.06)	0.215	0.92 (0.78, 1.10)	0.370
Obesity 2	1.00 (0.81, 1.25)	0.984	1.18 (0.91, 1.52)	0.207
Obesity 3	0.86 (0.41, 1.78)	0.685	1.40 (0.64, 3.06)	0.403
Comorbidities (n)	1.56 (1.49, 1.63)	< 0.001	1.35 (1.28, 1.43)	< 0.001
Shock	3.85 (2.52, 5.87)	< 0.001	3.75 (2.32, 6.08)	< 0.001
Oxygen saturation (%)	0.88 (0.86, 0.89)	< 0.001	0.93 (0.91, 0.95)	< 0.001
Lymphocyte (%)	0.91 (0.90, 0.91)	< 0.001	0.92 (0.92, 0.93)	< 0.001

*CI* confidence interval, *DIC* disseminated intravascular coagulation, *MODS* multiple organ dysfunction syndrome, *n* number, *OR* odds ratio. Body mass index (reference = normal).

**Table 4:** Logistic regression analysis for in-hospital death in patients with a possible DIC diagnosis on any of the days 1, 4, 8, and 15.

Parameters	Crude OR (95% CI)	*p*-value	Adjusted OR (95% CI)	*p*-value
DIC	7.26 (4.95,10.66)	< 0.001	4.76 (3.07,7.39)	< 0.001
Age (years)	1.05 (1.04,1.06)	< 0.001	1.07 (1.05,1.09)	< 0.001
Male sex	1.10 (0.79,1.53)	0.564	1.10 (0.73,1.65)	0.645
Body mass index				
Underweight	0.72 (0.37,1.40)	0.339	0.59 (0.28,1.27)	0.177
Obesity 1	0.94 (0.66,1.32)	0.708	1.42 (0.94,2.15)	0.094
Obesity 2	0.58 (0.32,1.04)	0.069	0.80 (0.39,1.66)	0.555
Obesity 3	1.52 (0.31,7.43)	0.603	4.53 (0.58,35.53)	0.151
Comorbidities (n)	1.34 (1.21,1.49)	< 0.001	1.17 (1.03,1.33)	0.019
Oxygen saturation (%)	0.98 (0.95,1.00)	0.101	1.03 (0.99,1.07)	0.104
Lymphocyte (%)	0.96 (0.94,0.97)	< 0.001	0.99 (0.98,1.01)	0.524
Complications (n)	2.37 (2.06,2.74)	< 0.001	2.36 (1.98,2.82)	< 0.001

*CI* confidence interval, *DIC* disseminated intravascular coagulation, *n* number, *OR* odds ratio. Body mass index (reference = normal).

### Model diagnostics

The *p*-values of the Hosmer–Lemeshow tests were > 0.05; none of the VIF values did not reach to 2.0, and Cook’s distances of all observed items were ˂0.1 (Supplementary Table 5).

## Discussion

This study confirmed that DIC incidence during the first two weeks after admission for COVID-19 using a nationwide database was approximately 10%, while DIC diagnosis on admission day was approximately 1%. DIC diagnosis upon admission showed moderate predictive ability for MODS and in-hospital death of patients with COVID-19. Patients with DIC showed a lower survival probability than non-DIC patients, and DIC diagnosis was independently associated with the occurrence of MODS and in-hospital death of patients with COVID-19. These results suggest that despite its low incidence, DIC notably influences the prognosis of patients with COVID-19.

The meta-analysis, summarizing 22 studies, confirmed that the pooled DIC incidence in COVID-19 during the very early pandemic from late December 2019 to February 2020 in China was 6.2%^20^. Systematic reviews and meta-analyses, including studies outside China, showed that the pooled DIC incidence in COVID-19 ranged from 3.0 to 4.3%^13,14^. These two meta-analyses included a very short period of patients with COVID-19 from the onset of the pandemic to the first several months. DIC incidence during the hospital stay in the present study was 10.9%. Our study presented an approximately two times higher DIC incidence as compared to the meta-analyses, which may be attributable to the study period from January 2020 to December 2021, when the SARS-CoV-2 delta variant was prevalent. It may also be influenced by multiple DIC diagnoses on several days during the hospital stay. Alternatively, the difference in diagnostic ability of diverse DIC diagnostic criteria used in the meta-analyses may also contribute to this variance. Helms et al.^21^ confirmed the marked difference in DIC incidence between JAAM (4%) and ISTH (0%) DIC scoring systems among patients with COVID-19. These results suggest that despite using variable diagnostic criteria for DIC, the approximate DIC incidence from the initial stage to late 2021 during the COVID-19 pandemic may be less than 10%.

Supplementary Tables 1 and 2 revealed that bacterial infections such as bacteremia and bacterial pneumonia are prominent, indicating that SARS-CoV-2-tiggered viral sepsis is not the only causative agent of DIC. Gradual increases in the diagnostic rate of DIC from 34.8% on day 1 to 40.3% on day 15 during the hospital stay may support this hypothesis. The present study revealed a very low incidence of DIC development (1.1%) on admission. During early SARS-CoV-2 infection, the influence of bacterial infection on COVID-19 pathogenesis is minimal. Therefore, the true incidence of DIC due to SARS-CoV-2-induced viral sepsis may be very low. However, marked incidences of gastrointestinal bleeding, deep vein thrombosis, and pulmonary thromboembolism and the effects of DIC diagnosis on admission to the poor prognosis of patients with COVID-19 should be considered.

Sepsis is a major cause of DIC. In bacterial sepsis, patients with DIC were more seriously ill and experienced complications, with a higher incidence of MODS, leading to a worse mortality rate than non-DIC patients^9^. Initial meta-analysis for DIC in COVID-19 demonstrated that the log risk ratio of DIC among non-survivors compared with survivors was 3.267, indicating that DIC is 26.2 times more likely to occur in non-survivors compared with survivors^20^. Another meta-analysis revealed that DIC correlated with COVID-19 disease severity and that death is more likely to be associated with DIC, suggesting that it also has crucial roles in SARS-CoV-2-induced viral sepsis similar to bacterial sepsis. This study confirmed the important role of DIC in the outcomes of patients with COVID-19 through more sophisticated methods. DIC diagnosed on admission, probably caused by SARS-CoV-2-induced viral sepsis, was independently associated with developing MODS on day 4 and in-hospital death of patients with COVID-19. The markedly lower survival probability of patients with DIC diagnosed on admission than those without DIC may be connected to the presentation of DIC on admission with MODS on day 4. Moderate but significant predictive performance of DIC score on admission for MODS and in-hospital mortality support these findings.

Initial antiviral innate immune responses are followed by inflammatory immune responses starting 6–8 days after SARS-CoV-2 infection. Once dysregulated, the inflammatory immune responses elicit life-threatening organ dysfunction during the first 2 weeks of infection^22^. Two large cohort studies showed that the differences in interleukin 6 levels, neutrophil counts, and D-dimer levels between survivors and non-survivors became evident around 10 days after SARS-CoV-2 infection^23,24^. A prospective autopsy study confirmed the coexistence of thrombosis and extensive inflammatory responses, presenting neutrophils and neutrophil extracellular traps associated with sporadic SARS-CoV-2 in multiple organs^25^. The median disease course of these patients was 22 days. These studies emphasized the significance of middle-stage pathologic inflammation but not SARS-CoV-2 itself in the pathogenesis of organ dysfunctions in COVID-19.

DIC is defined as dysregulated inflammatory and coagulofibrinolytic responses to insults. Despite the difficulty of eliminating the influences of bacterial infections, pathological inflammation after SARS-CoV-2 infection may lead to DIC diagnosed on days 1 to 15. Patients with DIC, especially those diagnosed on days 8 and 15, showed lower survival probability than those diagnosed on days 1 and 4 and non-DIC patients. This is consistent with the study of Tang et al.^12^, wherein coagulation and fibrinolysis parameters clearly deteriorated 7 days after admission, and non-survivors showed high DIC incidence. The present study confirmed that DIC diagnosis during the first 2 weeks after admission was independently associated with in-hospital death of patients with COVID-19, which supports the results of Tang et al. Hence, both early and late-stage DIC may be associated with poor prognosis of patients with COVID-19.

This study is limited by its retrospective design, including missing values and conduction within a single country, which may restrict the global generalization of the obtained results. Despite the adjusting with comorbidities, definitive effects of hospital-related bacterial infection on developing DIC could not be clearly excluded. Besides the major confounders, such as age and sex, the other confounders that affect the results should be considered. The patients who were discharged from hospitals before day 15 may also influence the interpretations of results. However, including a larger cohort of patients and prospectively collecting continuous data for DIC diagnosis and the evaluation of the diagnostic statistics may support the robustness of the used model and the results of this study.

## Conclusions

This study demonstrated DIC incidence in COVID-19 using a nationwide large-scale database. DIC incidence on admission was 1.1%, which increased to 10.9% during the first 2 weeks after admission. DIC diagnosed on admission presented with low survival probability and was independently associated with MODS on day 4 and in-hospital death of patients with COVID-19. DIC diagnosed on any of the days from 1 to 15, especially on days 8 and 15, had a lower survival probability than those without DIC and was independently associated with in-hospital death of patients with COVID-19. This indicates that despite its low incidence, both early- and late-stage DICs, especially DIC diagnosed within 1–2 weeks after admission, are crucial for the poor prognosis of patients with COVID-19.

### Supplementary Information

Supplementary Information 1.

Supplementary Information 2.

Supplementary Information 3.

Supplementary Information 4.

Supplementary Information 5.

Supplementary Information 6.

Supplementary Information 7.

## Data Availability

Data on an individual level are shared with limitation to participating healthcare facilities through applications to COVIREGI-JP.
