# Supplementary material for: Disseminated intravascular coagulation is associated with poor prognosis in patients with COVID-19
Source: Sci Rep. 2024 May 30;14:12443. doi: 10.1038/s41598-024-63078-9 (PMC11139854; doi:10.1038/s41598-024-63078-9)
Supplement: Supplementary file 1 — Supplementary Information 1. [file 41598_2024_63078_MOESM1_ESM.docx]

**Supplementary Figure 1.** Algorithm and scoring system for the Japanese Association for Acute Medicine (JAAM) DIC diagnostic criteria.
