# Supplementary figures and images for: Disseminated intravascular coagulation is associated with poor prognosis in patients with COVID-19
Source: Sci Rep. 2024 May 30;14:12443. doi: 10.1038/s41598-024-63078-9 (PMC11139854; doi:10.1038/s41598-024-63078-9)

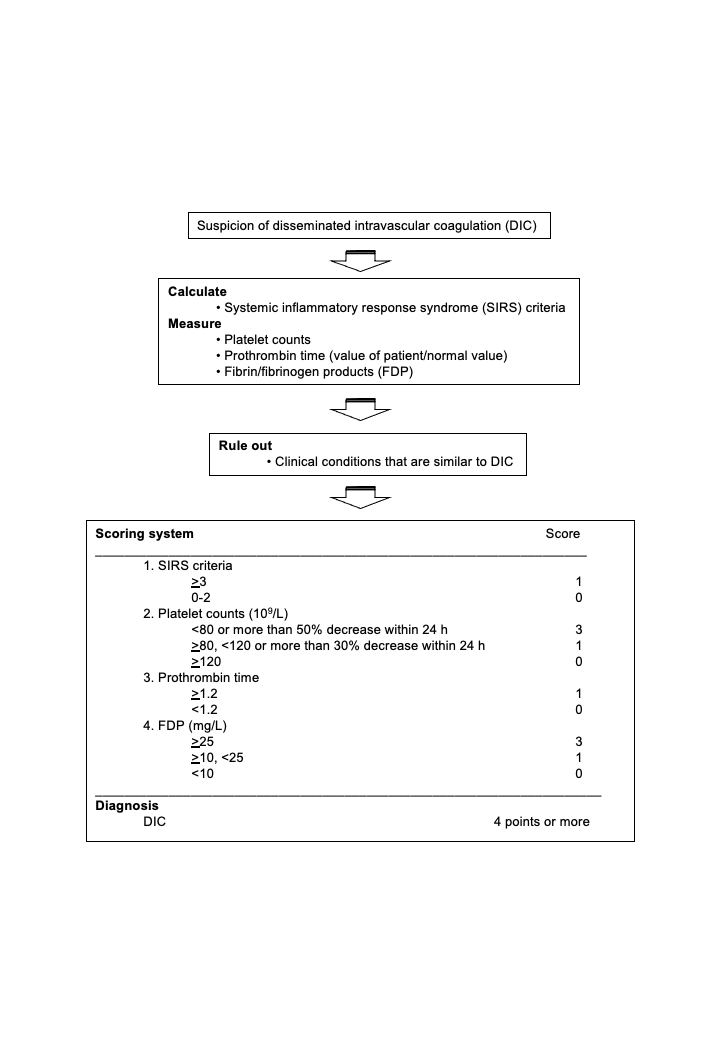

Supplement: Supplementary file 2 — Supplementary Information 2. [file 41598_2024_63078_MOESM2_ESM.tiff]
