# Supplementary material for: Disseminated intravascular coagulation is associated with poor prognosis in patients with COVID-19
Source: Sci Rep. 2024 May 30;14:12443. doi: 10.1038/s41598-024-63078-9 (PMC11139854; doi:10.1038/s41598-024-63078-9)
Supplement: Supplementary file 3 — Supplementary Information 3. [file 41598_2024_63078_MOESM3_ESM.docx]

**Supplementary Table 1.** Comorbidities and complications of the patients with a possible DIC diagnosis on admission (day 1)

|  | **All patients (n=23054)** | **Non-DIC (n=22790)** | **DIC (n=264)** | ***p*-value** |
| --- | --- | --- | --- | --- |
| **Comorbidities, n (%)** |  |  |  |  |
| Old myocardial infarction | 433 (1.9) | 420 (1.8) | 13 (4.9) | 0.002 |
| Congestive heart failure | 634 (2.8) | 621 (2.7) | 13 (4.9) | 0.037 |
| Cerebrovascular disease | 1525 (6.6) | 1489 (6.5) | 36 (13.6) | <0.001 |
| Dementia | 2102 (9.1) | 2056 (9.0) | 46 (17.4) | <0.001 |
| Chronic lung disease | 368 (1.6) | 357 (1.6) | 11 (4.2) | 0.004 |
| Bronchial asthma | 1304 (5.7) | 1295 (5.7) | 9 (3.4) | 0.138 |
| Mild liver disease | 555 (2.4) | 541 (2.4) | 14 (5.3) | 0.007 |
| Moderate to severe liver disease | 81 (0.4) | 73 (0.3) | 8 (3.0) | <0.001 |
| Mild diabetes mellitus | 3910 (17.0) | 3850 (16.9) | 60 (22.7) | 0.016 |
| Severe diabetes mellitus | 598 (2.6) | 581 (2.6) | 17 (6.4) | 0.001 |
| Obesity | 1589 (6.9) | 1574 (6.9) | 15 (5.7) | 0.540 |
| Moderate to severe kidney disease | 409 (1.8) | 396 (1.7) | 13 (4.9) | 0.001 |
| Maintenance hemodialysis | 249 (1.1) | 243 (1.1) | 6 (2.3) | 0.068 |
| Solid cancer | 820 (3.6) | 796 (3.5) | 24 (9.1) | <0.001 |
| Leukemia | 50 (0.2) | 46 (0.2) | 4 (1.5) | 0.003 |
| Lymphoma | 84 (0.4) | 83 (0.4) | 1 (0.4) | 0.621 |
| Metastatic solid cancer | 155 (0.7) | 146 (0.6) | 9 (3.4) | <0.001 |
| Collagen disease | 327 (1.4) | 324 (1.4) | 3 (1.1) | 1 |
| Human immunodeficiency virus infection | 43 (0.2) | 43 (0.2) | 0 (0.0) | 1 |
| Chronic obstructive pulmonary disease | 696 (3.0) | 679 (3.0) | 17 (6.4) | 0.003 |
| Hypertension | 7983 (34.6) | 7873 (34.6) | 110 (41.7) | 0.019 |
| Dyslipidemia | 3850 (16.7) | 3812 (16.7) | 38 (14.4) | 0.361 |
| **Complications, n (%)** |  |  |  |  |
| Bacterial pneumonia | 1565 (6.8) | 1504 (6.6) | 61 (23.1) | <0.001 |
| Acute respiratory distress syndrome | 1562 (6.8) | 1498 (6.6) | 64 (24.2) | <0.001 |
| Severity of acute respiratory distress syndrome |  |  |  |  |
| Mild | 283 (18.4) | 272 (18.4) | 11 (17.7) |  |
| Moderate | 578 (37.5) | 557 (37.7) | 21 (33.9) |  |
| Severe | 679 (44.1) | 649 (43.9) | 30 (48.4) | 0.796 |
| Meningitis | 12 (0.1) | 12 (0.1) | 0 (0.0) | 1 |
| Cerebrovascular disease | 100 (0.4) | 91 (0.4) | 9 (3.4) | <0.001 |
| Deep vein thrombosis | 207 (0.9) | 192 (0.8) | 15 (5.7) | <0.001 |
| Myocarditis/Pericarditis/Cardiomyopathy | 26 (0.1) | 24 (0.1) | 2 (0.8) | 0.035 |
| Myocardial ischemia | 52 (0.2) | 46 (0.2) | 6 (2.3) | <0.001 |
| Bacteremia | 298 (1.3) | 282 (1.2) | 16 (6.1) | <0.001 |
| Gastrointestinal bleeding | 157 (0.7) | 148 (0.6) | 9 (3.4) | <0.001 |
| Pulmonary thromboembolism | 92 (0.4) | 85 (0.4) | 7 (2.7) | <0.001 |

DIC, disseminated intravascular coagulation; n, number.
