# Supplementary material for: Disseminated intravascular coagulation is associated with poor prognosis in patients with COVID-19
Source: Sci Rep. 2024 May 30;14:12443. doi: 10.1038/s41598-024-63078-9 (PMC11139854; doi:10.1038/s41598-024-63078-9)
Supplement: Supplementary file 4 — Supplementary Information 4. [file 41598_2024_63078_MOESM4_ESM.docx]

**Supplementary Table 2.** Comorbidities and complications of the patients with a possible DIC diagnosis on any of the days 1, 4, 8, and 15

|  | **All patients (n=1654)** | **Non-DIC (n=1473)** | **DIC (n=181)** | ***p*-value** |
| --- | --- | --- | --- | --- |
| **Comorbidities n (%)** |  |  |  |  |
| Old myocardial infarction | 67 (4.1) | 52 (3.5) | 15 (8.3) | 0.005 |
| Congestive heart failure | 73 (4.4) | 64 (4.3) | 9 (5.0) | 0.700 |
| Cerebrovascular disease | 171 (10.3) | 148 (10.0) | 23 (12.7) | 0.300 |
| Dementia | 182 (11.0) | 168 (11.4) | 14 (7.7) | 0.165 |
| Chronic lung disease | 47 (2.8) | 39 (2.6) | 8 (4.4) | 0.230 |
| Bronchial asthma | 91 (5.5) | 85 (5.8) | 6 (3.3) | 0.225 |
| Mild liver disease | 71 (4.3) | 61 (4.1) | 10 (5.5) | 0.434 |
| Moderate to severe liver disease | 9 (0.5) | 6 (0.4) | 3 (1.7) | 0.066 |
| Mild diabetes mellitus | 418 (25.3) | 371 (25.2) | 47 (26.0) | 0.856 |
| Severe diabetes mellitus | 93 (5.6) | 76 (5.2) | 17 (9.4) | 0.026 |
| Obesity | 123 (7.4) | 112 (7.6) | 11 (6.1) | 0.549 |
| Moderate to severe kidney disease | 66 (4.0) | 53 (3.6) | 13 (7.2) | 0.027 |
| Maintenance hemodialysis | 44 (2.7) | 29 (2.0) | 15 (8.3) | <0.001 |
| Solid cancer | 87 (5.3) | 76 (5.2) | 11 (6.1) | 0.596 |
| Leukemia | 6 (0.4) | 5 (0.3) | 1 (0.6) | 0.502 |
| Lymphoma | 16 (1.0) | 13 (0.9) | 3 (1.7) | 0.407 |
| Metastatic solid cancer | 20 (1.2) | 15 (1.0) | 5 (2.8) | 0.059 |
| Collagen disease | 46 (2.8) | 39 (2.6) | 7 (3.9) | 0.336 |
| Human immunodeficiency virus infection | 2 (0.1) | 2 (0.1) | 0 (0.0) | 1 |
| Chronic obstructive pulmonary disease | 96 (5.8) | 80 (5.4) | 16 (8.8) | 0.089 |
| Hypertension | 817 (49.4) | 735 (49.9) | 82 (45.3) | 0.270 |
| Dyslipidemia | 408 (24.7) | 372 (25.3) | 36 (19.9) | 0.121 |
| **Complications n (%)** |  |  |  |  |
| Bacterial pneumonia | 418 (25.3) | 334 (22.7) | 84 (46.4) | <0.001 |
| Acute respiratory distress syndrome | 457 (27.6) | 358 (24.3) | 99 (54.7) | <0.001 |
| Severity of acute respiratory distress syndrome |  |  |  |  |
| Mild | 165 (36.7) | 133 (37.7) | 32 (33.0) |  |
| Moderate | 44 (9.8) | 38 (10.8) | 6 (6.2) |  |
| Severe | 241 (53.6) | 182 (51.6) | 59 (60.8) | 0.213 |
| Meningitis | 4 (0.2) | 3 (0.2) | 1 (0.6) | 0.371 |
| Stroke | 20 (1.2) | 11 (0.7) | 9 (5.0) | <0.001 |
| Deep vein thrombosis | 71 (4.3) | 57 (3.9) | 14 (7.7) | 0.030 |
| Myocarditis/Pericarditis/Cardiomyopathy | 6 (0.4) | 3 (0.2) | 3 (1.7) | 0.020 |
| Myocardial ischemia | 19 (1.1) | 15 (1.0) | 4 (2.2) | 0.146 |
| Bacteremia | 122 (7.4) | 83 (5.6) | 39 (21.5) | <0.001 |
| Gastrointestinal bleeding | 56 (3.4) | 40 (2.7) | 16 (8.8) | <0.001 |
| Pulmonary thromboembolism | 30 (1.8) | 24 (1.6) | 6 (3.3) | 0.131 |

DIC, disseminated intravascular coagulation, n, number.
