# Supplementary material for: Disseminated intravascular coagulation is associated with poor prognosis in patients with COVID-19
Source: Sci Rep. 2024 May 30;14:12443. doi: 10.1038/s41598-024-63078-9 (PMC11139854; doi:10.1038/s41598-024-63078-9)
Supplement: Supplementary file 5 — Supplementary Information 5. [file 41598_2024_63078_MOESM5_ESM.docx]

**Supplementary Table 3**. Stepwise logistic regression analysis for in-hospital death and MODS on day 4 in patients with a possible DIC diagnosis on admission (day 1)

| **Parameters** | **Crude OR (95%CI)** | ***p*-value** | **Adjusted OR (95%CI)** | ***p*-value** |
| --- | --- | --- | --- | --- |
| **In-hospital death** |  |  |  |  |
| DIC | 7.56 (5.39,10.61) | <0.001 | 2.02 (1.32,3.09) | 0.001 |
| Age (years) | 1.09 (1.08,1.09) | <0.001 | 1.09 (1.09,1.10) | <0.001 |
| Male gender | 1.19 (1.03,1.37) | 0.017 | 1.60 (1.35,1.90) | <0.001 |
| Comorbidities (n) | 1.71 (1.64,1.79) | <0.001 | 1.21 (1.14,1.29) | <0.001 |
| Shock | 2.95 (1.86,4.67) | <0.001 | 1.68 (0.97,2.91) | 0.066 |
| Oxygen saturation (%) | 0.87 (0.86,0.89) | <0.001 | 0.95 (0.94,0.97) | <0.001 |
| Lymphocyte (%) | 0.91 (0.91,0.92) | <0.001 | 0.96 (0.96,0.97) | <0.001 |
| Complications (n) | 4.30 (3.97,4.66) | <0.001 | 3.07 (2.81,3.36) | <0.001 |
| **MODS on day 4** |  |  |  |  |
| DIC | 7.21 (5.02,10.36) | <0.001 | 3.12 (2.07,4.68) | <0.001 |
| Age (years) | 1.04 (1.03,1.04) | <0.001 | 1.03 (1.02,1.03) | <0.001 |
| Male gender | 1.41 (1.23,1.63) | <0.001 | 1.49 (1.27,1.74) | <0.001 |
| Body mass index |  |  |  |  |
| Underweight | 1.67 (1.33,2.09) | <0.001 | 1.39 (1.08,1.79) | 0.010 |
| Obesity 1 | 0.91 (0.77,1.06) | 0.215 | 0.92 (0.78,1.1) | 0.370 |
| Obesity 2 | 1.00 (0.81,1.25) | 0.984 | 1.18 (0.91,1.52) | 0.207 |
| Obesity 3 | 0.86 (0.41,1.78) | 0.685 | 1.40 (0.64,3.06) | 0.403 |
| Comorbidities (n) | 1.56 (1.49,1.63) | <0.001 | 1.35 (1.28,1.43) | <0.001 |
| Shock | 3.85 (2.52,5.87) | <0.001 | 3.75 (2.32,6.08) | <0.001 |
| Oxygen saturation (%) | 0.88 (0.86,0.89) | <0.001 | 0.93 (0.91,0.95) | <0.001 |
| Lymphocyte (%) | 0.91 (0.90,0.91) | <0.001 | 0.92 (0.92,0.93) | <0.001 |

CI, confidence interval; DIC, disseminated intravascular coagulation; n, number; MODS, multiple organ dysfunction syndrome; n, number; OR, odds ratio. Body mass index (reference=normal)
