# Supplementary material for: Disseminated intravascular coagulation is associated with poor prognosis in patients with COVID-19
Source: Sci Rep. 2024 May 30;14:12443. doi: 10.1038/s41598-024-63078-9 (PMC11139854; doi:10.1038/s41598-024-63078-9)
Supplement: Supplementary file 6 — Supplementary Information 6. [file 41598_2024_63078_MOESM6_ESM.docx]

**Supplementary Table 4.** Stepwise logistic regression analysis for in-hospital death in patients with a possible DIC diagnosis on any of the days 1, 4, 8, and 15

| **Parameters** | **Crude OR (95% CI)** | ***p*-value** | **Adjusted OR (95%CI)** | ***p*-value** |
| --- | --- | --- | --- | --- |
| DIC | 7.26 (4.95,10.66) | <0.001 | 4.87 (3.15,7.54) | <0.001 |
| Age (years) | 1.05 (1.04,1.06) | <0.001 | 1.07 (1.05,1.09) | <0.001 |
| Body mass index |  |  |  |  |
| Underweight | 0.72 (0.37,1.40) | 0.339 | 0.59 (0.28,1.27) | 0.178 |
| Obesity 1 | 0.94 (0.66,1.32) | 0.708 | 1.42 (0.94,2.15) | 0.095 |
| Obesity 2 | 0.58 (0.32,1.04) | 0.069 | 0.79 (0.39,1.63) | 0.526 |
| Obesity 3 | 1.52 (0.31,7.43) | 0.603 | 4.35 (0.56,33.60) | 0.159 |
| Comorbidities (n) | 1.34 (1.21,1.49) | <0.001 | 1.17 (1.03,1.33) | 0.018 |
| Oxygen saturation (%) | 0.98 (0.95,1.00) | 0.101 | 1.03 (0.99,1.07) | 0.118 |
| Complications (n) | 2.37 (2.06,2.74) | <0.001 | 2.40 (2.03,2.85) | <0.001 |

CI, confidence interval; DIC, disseminated intravascular coagulation; n, number; OR, odds ratio. Body mass index (reference=normal)
