# Supplementary material for: Disseminated intravascular coagulation is associated with poor prognosis in patients with COVID-19
Source: Sci Rep. 2024 May 30;14:12443. doi: 10.1038/s41598-024-63078-9 (PMC11139854; doi:10.1038/s41598-024-63078-9)
Supplement: Supplementary file 7 — Supplementary Information 7. [file 41598_2024_63078_MOESM7_ESM.docx]

Supplementary Table 5 Diagnostic statistics for model fit

|  | Hosmer-Lemshow p-value | Predictive accuracy | Variance Inflation Factor | Maximum Cook’s distance |
| --- | --- | --- | --- | --- |
| Table 3 |  |  |  |  |
| In-hospital mortality | 0.5026 | 95.5 | < 2.0 | 0.040 |
| MODS | 0.0546 | 90.2 | < 2.0 | 0.013 |
| Table 4 |  |  |  |  |
| In-hospital mortality | 0.0605 | 86.7 | < 2.0 | 0.094 |

MODS, multiple organ dysfunction syndrome.
